# Health benefits of *Bifidobacterium animalis* subsp. *lactis* BB-12 in infants and children: a mini-review

**DOI:** 10.3389/fmicb.2026.1773473

**Published:** 2026-02-19

**Authors:** Carlos Patricio Acosta Rodríguez Bueno, Ailim Carias Domínguez, Denis Guyonnet, Etienne Pouteau

**Affiliations:** 1 Hospital Infantil de México Federico Gómez, Ciudad de México, Mexico; 2 Fundación Santa Fe de Bogotá, Bogotá, Colombia; 3 Opella, Neuilly-Sur-Seine, France

**Keywords:** BB-12, *Bifidobacterium*, digestive health, gut microbiota, gut-brain disorders, probiotics in children

## Abstract

The colonization of the infant gut microbiome during the critical window of 0–3 years is influenced by a multitude of prenatal, environmental and host factors, and may be crucial for life-long health. The infant gut microbiome is highly dynamic, with bifidobacteria*-*dominance generally established during birth and lactation, followed by transition to a more stable and adult-like composition reached around 3 years of age. Bifidobacteria-dominance in infancy is considered protective as they not only display both anti-inflammatory and immunomodulatory effects but also foster the establishment of other beneficial species via cross-feeding interactions. As predominance of bifidobacteria is considered the marker of a healthy breastfed infant, the use of bifidobacteria-based probiotics for the prevention of gut dysbiosis and related conditions has been investigated. This clinically oriented summary highlights the unmet research needs of *Bifidobacterium animalis* subsp. *lactis*, BB-12^®^ (BB-12), a well-studied probiotic added to baby formulas, dietary supplements, and fermented milk products; several potentially beneficial attributes, including acid and bile tolerance, strong adherence properties, pathogen inhibition, and immune modulation are considered. Clinical studies have demonstrated the safety and beneficial effects of BB-12 in infants and children across multiple disorders and highlight the need for improved clinical and regulatory guidelines.

## 1 Introduction

The gut microbiota is a diverse microbial community that colonizes the human gastrointestinal (GI) tract. The genetic and functional profile of microbial species is termed the gut microbiome (Maynard et al., 2012). Healthy development and composition of the gut microbiome in infants may be crucial for normal gut physiological functions (e.g., intestinal permeability and motility), energy production, neurocognitive and immunological development, and long-term health (Yang et al., 2016). Multiple factors influence development of the infant gut microbiome, including maternal diet, antibiotic use, infections during pregnancy, delivery type, and breastfeeding and weaning (Suarez-Martinez et al., 2023; Anania et al., 2021; Argentini et al., 2022).

At birth, the infant gut microbiome exhibits low diversity, comprised of clostridia, bacteroides and bifidobacteria, shifting within 7 days to predominately (∼40%–80%) infant-type species *Bifidobacterium* (*B. longum* subsp. *infantis, B. bifidum, B. breve and B. longum* subsp. *Longum*), *Escherichia*/*Shigella* and *Veillonella* (Picard et al., 2005; Arrieta et al., 2014; Lin et al., 2022; Barker-Tejeda et al., 2024). Intestinal diversity increases throughout the first 3 years of life, progressing to a stable, adult-like gut microbiota, with homeostasis (eubiosis) established (DeGruttola et al., 2016; Yang et al., 2016; Miqdady et al., 2020; Akagawa et al., 2021). The establishment of a favorable gut microbiota during infancy is essential, as early life dysbiosis (imbalanced and disrupted gut microbiota) is associated with poor health later in life (Akagawa et al., 2021).

Prevalence of *Bifidobacterium* species in the GI tract is considered the landmark of a healthy infant, as they convey both anti-inflammatory and immunomodulatory effects while fostering the establishment of other beneficial species via cross-feeding interactions (Saturio et al., 2021; Anania et al., 2021; Argentini et al., 2022; Strisciuglio et al., 2023). As such, use of bifidobacteria-based probiotics for the maintenance of a balanced microbiota and, therefore, prevention of gut dysbiosis, is of scientific interest, leading to the inclusion of bifidobacteria into guidelines and probiotic formulations for pediatric populations (Saturio et al., 2021; Mercer and Arrieta, 2023).

This clinically oriented mini-review summarizes the key attributes and the role of *Bifidobacterium* in eubiosis and describes the clinical evidence supporting the use of the BB-12 strain in pediatric populations.

## 2 Factors impacting the infant gut microbiome

Multiple environmental and host factors guide the development of the infant gut microbiome. For example, among prenatal factors (Penders et al., 2006; Koleva et al., 2015; Stuivenberg et al., 2022), delivery mode is a major determinant, with the gut microbiota of neonates delivered vaginally typically dominated by *Lactobacillus*, *Prevotella*, and *Sneathia* genera, reflecting the microbiota of the maternal birth canal or intestinal tract. Conversely, the gut microbiota of newborns delivered by cesarean is influenced by maternal skin microbiota, with a higher proportion of *Staphylococcus*, *Corynebacterium*, and *Propionobacterium* species, potentially increasing susceptibility to infections and allergies (Al Bander et al., 2020; Akagawa et al., 2021). Gestational age has been shown to impact microbial colonization, with pre-term infants exhibiting lower diversity and reduced *Bifidobacterium* species compared with full-term infants, resulting in immature gut barrier function and immunity, as *Bifidobacterium* play a crucial role in maintaining and enhancing health (Jia et al., 2022; Akagawa et al., 2021). Other factors, including maternal diet, use of antibiotics or antiacid medication, also contribute to shaping an infant’s gut microbiome (Akagawa et al., 2021). Geographical location can also influence gut colonization (Stewart et al., 2018), with a decrease in bifidobacteria, specifically *Bifidum longum* subsp. *infantis* (*B. infantis*), observed in infants from industrialized countries compared with non-industrialized countries (Olm et al., 2022; Saturio et al., 2021). Establishment of healthy gut microbiota is important for general health, including the development and maturation of the immune system. Gut dysbiosis has been implicated in many gut-brain axis disorders experienced by infants and toddlers, such as regurgitation, constipation and infantile colic, which can lead to non-optimal development, disrupt digestion and lower quality of life (DeGruttola et al., 2016; Sarkar et al., 2021; García-Santos et al., 2023; Kwiatkowska et al., 2024). Furthermore, early dysbiosis is considered a causal factor in multiple diseases later in life, with decreased bifidobacteria a potential risk marker for metabolic-related diseases (e.g., obesity, diabetes, atopic conditions and neurodevelopment disorders) (Sarkar et al., 2021; Saturio et al., 2021).

## 3 Bifidobacterium

*Bifidobacterium*, a Gram-positive anaerobic bacterium first isolated from the feces of a breastfed infant, is non-spore-forming, non-motile and able to produce lactic acid (Jungersen et al., 2014; Saturio et al., 2021). Early dominance in the infant’s gut is considered protective (Saturio et al., 2021), as bifidobacteria exhibit many beneficial properties, including anti-inflammatory effects, enhancement of gut barrier function, pathogen inhibition, nutrient absorption and immune modulation (Akagawa et al., 2021; Saturio et al., 2021; Fanning et al., 2012; Maslowski et al., 2009).

Several compounds that promote bifidobacteria growth have been identified in breast milk (Lawson et al., 2020). The introduction of complementary feeding at ∼6 months of life decreases the natural levels of *Bifidobacterium* (Bergström et al., 2014); however, the influence of early *Bifidobacterium* dominance on health persists, providing long-term health benefits, such as improved vaccine response (Huda et al., 2014), and reduced risks of obesity (Kalliomäki et al., 2008) and allergy (Sjögren et al., 2009). In comparison, formula-fed infants exhibit increased *Enterobacteriaceae, Bacteroidaceae and Clostridiaceae* and greater microbial diversity (Chong et al., 2022).

**FIGURE 1:**
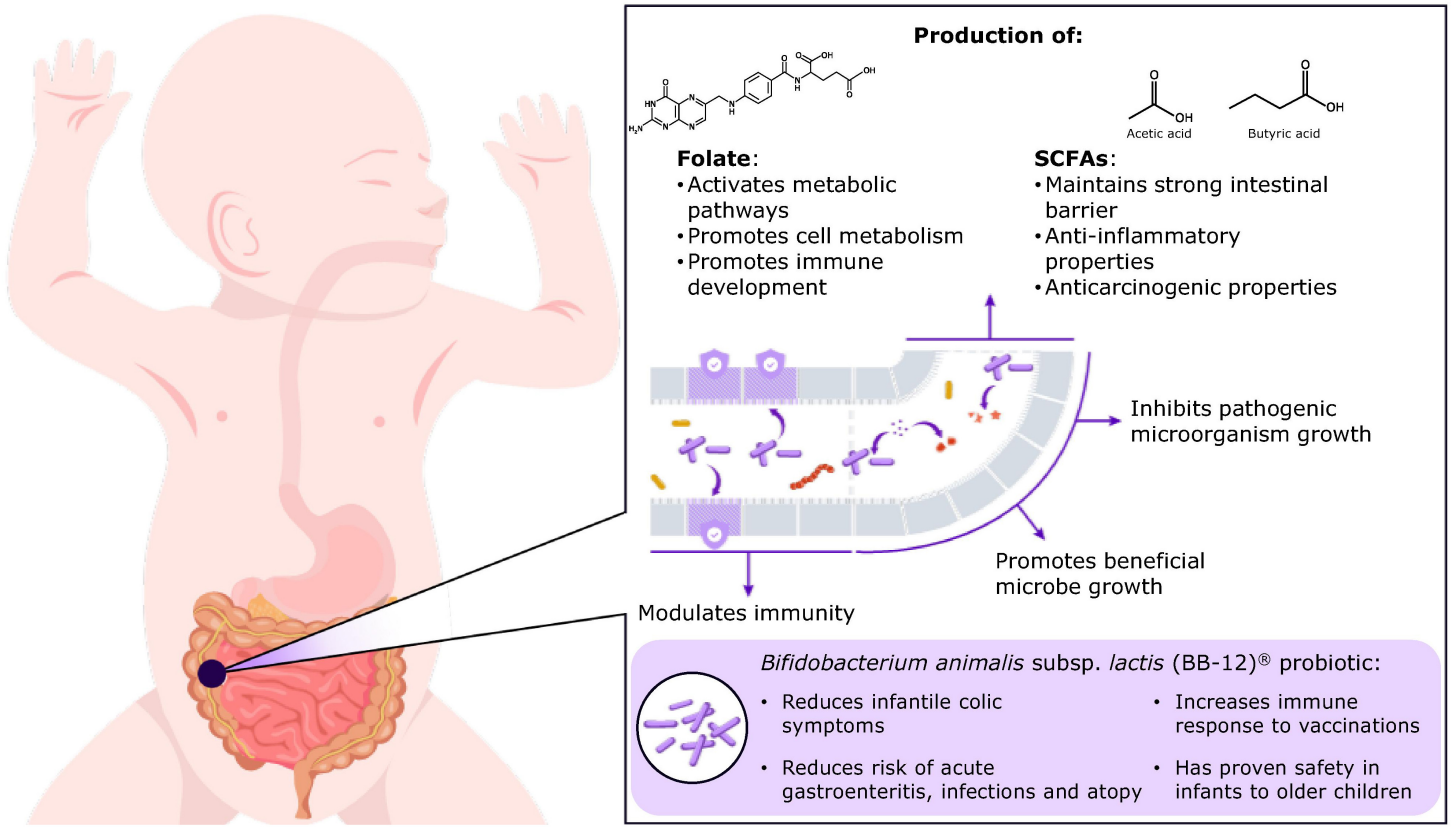
Overview of the influence of bifidobacteria on the development of the infant gut and clinical indications for BB-12^®^. SCFA, short-chain fatty acids.

The beneficial effects of *Bifidobacterium* are a consequence of multiple biological functions (Figure 1). The metabolism of human milk oligosaccharides (HMOs) by bifidobacteria produces substances, including short-chain fatty acids (SCFAs) (e.g., acetate, propionate and butyrate), that support the growth of other health-promoting microbes through cross-feeding (Lawson et al., 2020), inhibit the growth of pathogens and other bacterial species by reducing luminal pH (Pokusaeva et al., 2011; Taft et al., 2018; Ríos-Covián et al., 2016), improve intestinal barrier function (Yoo et al., 2020; Stuivenberg et al., 2022; Lin et al., 2022), and serve as an energy source for colonocytes (Rivière et al., 2016; Lin et al., 2022). SCFAs secreted by bifidobacteria are also implicated in host metabolism (Ríos-Covián et al., 2016) and early neurocognitive development, including brain development, neuronal firing and the expression of neurotransmitters and receptors (Rivière et al., 2016; Lin et al., 2022; Yang et al., 2016). For example, acetate has been shown to directly modulate hypothalamic neuron activation, implicating bifidobacteria in body weight regulation (Lin et al., 2022; Hernandez et al., 2019). Further, SCFAs influence the sympathetic and enteric nervous systems through the gut-brain axis (García-Santos et al., 2023; Han et al., 2021).

Bifidobacteria may improve depressive-like symptoms and mood via serotonin production through tryptophan pathway modulation, as shown in pre-clinical studies (Tian et al., 2019), potentially categorizing Bifidobacteria as psychobiotics. Additionally, serotonin (5-HT)-enriched neonatal intestines promote regulatory T cell differentiation and tolerance to dietary antigens, improving immunity (Sanidad et al., 2024). Wang et al. (2025) also linked the BB-12 strain to reduced clinical food intolerance incidence. Bifidobacteria have the enhanced ability to adhere to the intestinal epithelium, and by competing for space and nutrients, they prevent the establishment of potentially pathogenic microbes and protect against intestinal infections (Walsh et al., 2023; Lawson et al., 2020; Stuivenberg et al., 2022; Taft et al., 2018).

Bifidobacteria influence neonatal immune system development directly or via metabolites (Lin et al., 2022), stimulating dendritic cells and immunoglobulin A (IgA), T lymphocyte development, and specific and non-specific antibody production (Ruiz et al., 2017; López et al., 2010; Lin et al., 2022, Lau et al., 2015). For example, butyrate exerts anti-inflammatory effects through the upregulation of interleukin (IL)-10, production of regulatory T cells, and inhibition of nuclear factor kappa-light-chain-enhancer of activated B cells (NF-κB) signaling (Saban Güler et al., 2025). Moreover, SCFA-mediated G-protein coupled receptor 43 signaling attenuates the secretion of pro-inflammatory cytokines (IL-6, IL-12, and tumor necrosis factor-α), essential in the prevention of colonic inflammation and related cancers (Singh et al., 2014; Vinolo et al., 2011; Yoo et al., 2020). Additionally, bifidobacteria may boost vaccine protection in infants by enhancing systemic and mucosal memory T-cell and antibody responses (Huda et al., 2014). Overall, with approximately 70%–80% of all immune cells located in the intestine, the interplay between intestinal function and immunity cannot be underestimated (Wiertsema et al., 2021).

### 3.1 *Bifidobacterium* in probiotics

Probiotics are live microorganisms that, when administered in adequate amounts, confer a health benefit on the host (Hill et al., 2014; Mao et al., 2021). Probiotics are considered safe for human consumption and represent one of the main strategies used to modulate gut microbiota, with *Bifidobacterium* and *Lactobacillus* widely used for their ability to prevent and treat multiple GI disorders (Holscher et al., 2012; Picard et al., 2005; Mao et al., 2021). Notably, supplementation of infant formula with probiotics (usually present in breast milk, specifically bifidobacteria) has been used to manage infant gut dysbiosis in premature infants and those delivered by cesarean section (Eor et al., 2023; Holscher et al., 2012). A few species of bifidobacteria (*Bifidobacterium adolescentis, Bifidobacterium animalis, Bifidobacterium bifidum, Bifidobacterium breve, and Bifidobacterium longum*) have been granted Qualified Presumption of Safety (QPS) by the European Food Safety Authority (EFSA) (Saturio et al., 2021).

### 3.2 *Bifidobacterium animalis* subsp. *lactis* BB-12

*Bifidobacterium* BB-12 (BB-12^®^), a catalase-negative, rod-shaped bacterium, classified as *Bifidobacterium animalis* subsp. *lactis* (Jungersen et al., 2014), has been widely used in baby formula, dietary supplements, and fermented milk products (Jungersen et al., 2014). It was granted QPS in 2007 and is recognized as safe by the Food and Drug Administration (FDA) (Us Food and Drug Administration [FDA], 2019; EFSA BIOHAZ Panel (EFSA Panel on Biological Hazards) et al., 2026). BB-12 was isolated based on several desirable probiotic characteristics (Jungersen et al., 2014).

It exhibits high gastric acid and bile tolerance, potentially via intracellular pH regulation through H^+^-ATPase induction, improving the chance of GI survival (Vernazza et al., 2006; Jungersen et al., 2014). BB-12 adapts to high bile salt concentrations in the small intestine via active bile salt hydrolase, ensuring its survival in the GI tract (Jungersen et al., 2014), with multiple studies confirming fecal recovery of BB-12 ≤ 2 weeks after supplementation (Jungersen et al., 2014; Vernazza et al., 2006).

BB-12 improves gut barrier function by regulating tight junctions, and preclinical studies have shown that its fermentation products increase trans-epithelial electrical resistance (Commane et al., 2005; Collins et al., 2025).

Although strong mucosal adherence is the primary characteristic of BB-12 responsible for pathogen inhibition, the exact mechanism remains unknown. BB-12 has been shown to produce inhibitory substances with antagonistic activity against pathogens like *Bacillus cereus, Clostridium difficile, Escherichia coli, Listeria monocytogenes, Pseudomonas aeruginosa, Shigella flexneri, Shigella sonnei*, and *Salmonella typhimurium* (Martins et al., 2009; Jungersen et al., 2014). Nutrient competition and depletion, and activation of the immune system by BB-12 supplementation may also contribute to pathogen inhibition.

Lastly, as BB-12 has been shown to interact with the immune system, primarily by inducing dendritic cell maturation and multiple anti-inflammatory cytokines (IL-10, IL-12, IFN-γ), supplementation may positively impact immune function (Jungersen et al., 2014; Collins et al., 2025). Due to the beneficial properties described above, BB-12 is one of the most widely studied probiotics, with clinical testing dating back to 1987 (Jungersen et al., 2014).

Mechanisms of action of BB-12 have been described in a recent review, including effect on gut-brain axis and SCFA (Collins et al., 2025).

### 3.3 Safety of BB-12

Bifidobacteria are generally considered non-pathogenic; nevertheless, the safety and tolerance of BB-12 have been extensively investigated in pediatric populations, with no safety concerns or adverse effects noted (Nocerino et al., 2020; Chouraqui et al., 2004; Taipale et al., 2016; Weizman et al., 2005; Mihatsch et al., 2010; Hojsak et al., 2016; Chen et al., 2021). BB-12 displays resistance to several antibiotics (e.g., cloxacillin and vancomycin), but the potential for transfer of antibiotic resistance is null as intrinsic resistance genes are devoid of mobile elements, confirming its safety (Mohan et al., 2006; Rozman et al., 2023).

### 3.4 Clinical efficacy of BB-12

A systematic review of studies implementing a randomized, blind, placebo-controlled design was performed to identify the efficacy of BB-12 for the management of digestive and immune disorders in pediatric populations (Table 1). The search was conducted on Medline in March 2025 using the keywords “blinded,” “randomized,” “human,” “BB-12,” “digestive,” “immune disorders,” and “children.” Though most studies focused on the treatment of infantile colic, the impact of BB-12 on other GI disorders and immunity has also been investigated.

**TABLE 1:** Summary of efficacy and safety *Bifidobacterium animalis* subsp. *lactis*, BB-12^®^ (BB-12) studies in infants and children.

References	Treatment	Daily dose (CFU)	Participants (N)	Study design and population	Aim	Clinical results
Wang et al., 2025	BB-12 vs. placebo	1 × 10^9^	71	A 1-month double-blind RCT in preterm infants	To evaluate intestinal metabolites and the levels of serum inflammatory markers	Infants receiving BB-12 had more amino acids, lower inflammatory markers and a lower incidence of feeding intolerance (*P* < 0.05)
Chen et al., 2021	BB-12 vs. placebo	1 × 10^9^	192	A 21-day double-blind RCT in breastfed Chinese infants aged < 12 weeks at enrollment	To assess the efficacy of BB-12 in the management of infantile colic and the rate of infants with a reduction of >50% of mean daily crying duration	A higher percentage of infants receiving BB-12 achieved a ≥ 50% reduction in daily crying/fussing after the 21-day (*P* < 0.001), with the mean number of crying episodes also reduced and the mean daily sleep duration increased
Nocerino et al., 2020	BB-12 vs. placebo	1 × 10^9^	80	A 28- day RCT in healthy infants, aged ≤ 7 weeks, with colic	To assess the rate of infants with a reduction of >50% of mean daily crying duration	A higher percentage of infants receiving BB-12 achieved a ≥ 50% reduction in daily crying duration, with the mean number of crying episodes also reduced and daily stool frequency decreased
Weizman et al., 2005	BB-12+ *L. reuteri* vs. placebo	1 × 10^7^	201	A 12-week double-blind RCT in healthy, full-term infants, aged 4–10 months, attending childcare centers	To compare the effect of two species of probiotic bacteria in preventing infection	Compared with control, BB-12 resulted in fewer febrile episodes, fewer and shorter episodes of diarrhea
Chouraqui et al., 2004	BB-12 vs. placebo	1.5 × 10^8^	90	A multicenter, double-blind RCT in infants aged < 8 months admitted to a residential center for at least 4 months	To assess the efficacy and tolerability of a milk formula supplemented with BB-12 in the prevention of acute diarrhea	Infants receiving BB-12 supplemented formula had fewer, shorter episodes of diarrhea compared with control. Overall, BB-12 reduced the risk of diarrhea by a factor of 1.9 (range, 1.33–2.6)
Saavedra et al., 2004	BB-12+ *S. thermophilus* vs. placebo	1 × 10^7^	118	A prospective, double-blind RCT in healthy infants aged 3–24 months; duration ranged between 17 and 565 days	To evaluate tolerance to formulas containing two species of probiotic supplementation and their effect on growth, general clinical status, and intestinal health	The supplemented formulas were Well-tolerated and associated with reduced frequency of colic or irritability (*P* < 0.001) and antibiotic use (*P* < 0.001) compared with control
Mohan et al., 2006	BB-12 vs. placebo	D1–3: 1.6 × 10^9^; >D4: 4.8 × 10^9^	69	A 21-day double-blind RCT in preterm infants with a gestational age < 37 weeks	To evaluate whether the supplementation of preterm infants with BB-12 results in the modification of gut microbiota to suppress the growth of potentially harmful bacteria	Compared with control, the number of bifidobacteria significantly increased (*P* < 0.001) with BB-12 supplementation, and lower viable counts of *Enterobacteriaceae* (*P* = 0.015) and *Clostridium spp.* (*P* = 0.014) were observed; however, supplementation did not reduce colonization of antibiotic-resistant organisms
Taipale et al., 2016	BB-12 vs. placebo	1 × 10^10^	109	Double-blind RCT in healthy infants aged 1 month until the age of 2 years	To investigate the impact of BB-12 on the risk of acute infectious diseases	Compared with control, infants receiving BB-12 experienced fewer respiratory tract infections (*P* = 0.033), but no significant difference in gastrointestinal symptoms, otitis media, or fever was observed
Holscher et al., 2012	BB-12 vs. placebo	1 × 10^6^	172	A 6-week double-blind RCT in healthy, full-term infants aged 6 weeks	To assess the effect of an infant starter formula containing BB-12 on intestinal immunity and inflammation	Among vaginally delivered infants, BB-12 increased fecal sIgA compared with control (*P* < 0.05). Anti-poliovirus-specific IgA concentration increased in all infants, regardless of the mode of delivery (*P* < 0.05), whereas antirotavirus-specific IgA increased in cesarean-delivered infants (*P* = 0.056)
Isolauri et al., 2000	BB-12 vs. LGG vs. placebo	BB-12: 1 × 10^9^; LGG: 3 × 10^8^	27	A 2-month, double-blind RCT in infants with early onset atopic eczema	To assess the potential of probiotics to control allergic inflammation at an early age	Compared with control, 2 months of supplementation resulted in a significant improvement in skin condition (*P* = 0.002), with the SCORAD score and the concentration of soluble CD4 in serum and eosinophilic protein X in urine decreased with both BB-12 and LGG
Kirjavainen et al., 2002	BB-12 vs. placebo	8 × 10^10^/kg body weight	21	A RCT in infants with early onset atopic eczema either highly sensitive or tolerant to extensively hydrolyzed whey formula	To characterize the relationship between gut microbes and the extent of allergic sensitization and to assess whether the efficacy of BB-12 supplementation could relate to modulation of the intestinal microbiota	Infants highly sensitized to EHF displayed greater numbers of lactobacilli/enterococci than tolerant infants. Serum total IgE concentration correlated with *E. coli* counts in all infants and with bacteroides counts in the highly sensitized infants, indicating the involvement of these bacteria in atopic sensitization. BB-12 supplementation reduced *E. coli* count and protected against an increase in bacteroides during weaning

CD4, cluster of differentiation 4; CFU, colony-forming units; EHF, extensively hydrolyzed whey formula; IgE, immunoglobulin E; LGG, *L.rhamnosus*; RCT, randomized controlled trial; SCORAD, SCORing Atopic Dermatitis; sIgA, secretory immunoglobulin A.

#### 3.4.1 Colic symptoms

Infantile colic, characterized by recurrent and prolonged periods of crying, fussing or irritability without evidence of cause or other clinical signs, affects ∼20% of newborns in the first 5 months (García-Santos et al., 2023; Banks et al., 2023). Compared with healthy infants, the gut microbiota of infants with colic is characterized by high levels of potentially pathogenic bacteria and decreased levels of *Bifidobacterium* and *Lactobacillus*, implicating gut dysbiosis in colic (García-Santos et al., 2023). This led to the investigation of probiotic supplementation as a potential therapeutic option.

A randomized, double-blind, placebo-controlled study suggested that 21 days of supplementation with BB-12 [1 × 10^9^ colony-forming units (CFU)] is an effective treatment for infantile colic (Chen et al., 2021). A significantly higher proportion of infants supplemented with BB-12 achieved ≥50% reduction in duration of crying and fussing (61.5% vs. 21.9%; *p* < 0.001), reduction in daily crying episodes (10.0 ± 3.0 to 5.0 ± 1.9 vs. 10.5 ± 2.6 to 7.5 ± 2.8; *p* < 0.001), and an increase in mean daily sleep duration (60.7 ± 104.0 vs. 31.9 ± 102.7 min/day; *p* < 0.001), compared with placebo, respectively (Chen et al., 2021). Furthermore, BB-12 supplementation increased health-related quality of life parameters for parents/caregivers with colicky infants, with higher scores for physical, emotional and social functioning noted compared with the placebo group (Chen et al., 2021). Another study found that 28 days of supplementation with BB-12 (1 × 10^9^ CFU) significantly reduced daily crying duration by over half (80% of infants vs. 32.5%; *p* < 0.0001) and daily crying episodes (−4.7 ± 3.4 vs. −2.3 ± 2.2; *p* = 0.001) compared with placebo (Nocerino et al., 2020). Additionally, increased bifidobacteria correlated with a reduction in crying time in responder infants (Nocerino et al., 2020). Lastly, another study confirmed that long-term consumption of formula supplemented with BB-12 and *Streptococcus thermophilus* (*Str thermophilus*) was well-tolerated and reduced the incidence of colic and irritability in infants (Saavedra et al., 2004). Overall, these studies support the use of BB-12 in the management of colic-related symptoms in infants (World Gastroenterology Organisation, 2023).

#### 3.4.2 Digestive health

BB-12 supports normal digestion (Jungersen et al., 2014), with studies showing that supplementation increases the proportion of beneficial bacteria in the gut whilst reducing the proportion of potentially pathogenic bacteria (Mättö et al., 2006; Hornef, 2015; Merenstein et al., 2021).

Studies have shown the benefit of BB-12 in reducing the incidence and severity of intestinal disorders in children (e.g., diarrhea, constipation and gastroesophageal reflux disease) (Saturio et al., 2021). Infants fed a formula containing BB-12 and *Lactobacillus reuteri* (SD 2112) experienced significantly fewer episodes of diarrhea (0.13 vs. 0.31), with shorter duration (0.37 vs. 0.59 days), compared with infants fed a control formula, respectively (Weizman et al., 2005). The diarrhea risk in infants fed BB-12-enriched formula decreased by a factor of 1.9 (range, 1.33–2.6) compared with control, suggesting that BB-12 might exert a protective effect against acute gastroenteritis (Chouraqui et al., 2004). A systematic review found that BB-12 may reduce the risk of necrotizing enterocolitis (NEC) through the modulation of systemic NF-κB-dependent inflammatory responses and reinforcement of gut barrier function and integrity (Beghetti et al., 2021; Morgan et al., 2020; García-Santos et al., 2023). The clinical effect of BB-12 in colic may also be due to a beneficial effect on the regulation of intestinal transit (Pitkala et al., 2007; World Gastroenterology Organisation, 2023). Additionally, a safety review of infants fed a symbiotic formula supplemented with BB-12 and fructo-oligosaccharides with lactose showed a significant decrease in episodes of functional constipation (3.2%), regurgitation (10.2%) and infantile crying and colic (10.5%) compared with historical prevalence (7.8%, 26.7%, and 17.7%, respectively) (Depoorter and Vandenplas, 2021).

#### 3.4.3 Immunity

Multiple studies have assessed the impact of BB-12 on the neonatal immune system. For example, one study found that BB-12 supplementation significantly reduced the number and frequency of respiratory tract infections (RTIs) during the first 2 years of life, compared with placebo (Taipale et al., 2016). Of note, one study showed conflicting results, with BB-12 not impacting RTI incidence (Hojsak et al., 2016).

Furthermore, several studies have implicated BB-12 in the modulation of the immune response to vaccination (Holscher et al., 2012; Huda et al., 2014; Rizzardini et al., 2012), with an abundance of bifidobacteria in early infancy associated with improved vaccine responsiveness (Huda et al., 2014). One study found that cesarean-delivered infants fed with a formula containing BB-12 displayed an increased immune response to poliovirus and rotavirus vaccination, predominantly mediated by fecal secretory IgA (Holscher et al., 2012). A similar effect was also shown in adults (20–60 years), with an increased adaptive immune response following influenza vaccination after BB-12 supplementation, and a significant increase in vaccine-specific IgG, IgG1, and IgG3 observed (Rizzardini et al., 2012).

BB-12 supplementation has also been shown to alleviate allergic inflammation in infants with early-onset atopic eczema (Isolauri et al., 2000; Kirjavainen et al., 2002). As the intestinal microbiota plays a role in the development of food allergies, the use of probiotic-based therapy has gained interest due to its immunomodulatory effects, including the production of T-helper (Th) 1 cells, development of tolerogenic dendritic cells and suppression of Th2 and IgE. Furthermore, enhanced gut barrier integrity leads to decreased accessibility of dietary antigens and, therefore, reduced allergen sensitization. Despite these benefits, the use of probiotics for the modulation of food allergies requires further investigation (García-Santos et al., 2023).

Lastly, BB-12 supplementation has been shown to alleviate inflammatory response in premature infants, thereby protecting the intestinal mucosa and promoting intestinal development. A study showed that 28 days of supplementation with BB-12 (1 × 10^9^ CFU) significantly increased amino-acid content in intestinal metabolic products (especially those responsible for gluconeogenesis), significantly reduced levels of serum inflammatory markers, including toll-like receptor 2, nuclear factor kappa B and tumor necrosis factor-α (*p* ≤ 0.05), and lowered incidence of feeding intolerance compared with placebo (18 [50%] vs. 27 [77.1%], respectively [*p* = 0.05]) (Wang et al., 2025).

## 4 Guidelines and recommendations

Despite the abundance of clinical studies reporting the safety and efficacy of BB-12, limited information on clinical indications, dosage, and duration of treatment in pediatric populations exists, with current recommendations providing levels of evidence for probiotic benefits only (García-Santos et al., 2023).

The European Society for Pediatric Gastroenterology Hepatology and Nutrition (ESPGHAN) recommends BB-12, alone or in combination, for the prevention of mild/severe NEC (Szajewska et al., 2023). However, since the level of evidence is low, the American Academy of Pediatricians advises against routine administration in preterm infants weighing less than 1,000 g due to lack of FDA quality regulation (Depoorter and Vandenplas, 2021).

There is moderate evidence for the use of BB-12 in the management of colic and colic-related symptoms, with guidelines available from the World Gastroenterology Organization and ESPGHAN (World Gastroenterology Organisation, 2023). BB-12 is also recommended for the treatment of gut-brain axis disorders by ESPGHAN and for the prevention of nosocomial diarrhea and acute infectious diarrhea by the Latin American Expert consensus group, but with only moderate certainty (Cruchet et al., 2015; Szajewska et al., 2023).

Regarding dosage and duration, it is recommended that probiotics be administered in line with relevant clinical studies, based on the treatment population and their risk of disease (van den Akker et al., 2020). For example, BB-12 at a dose of 1 × 10^8^ CFU/day for 21–28 days is recommended for the management of infantile colic, but a higher dose (3.0–3.5 × 10^8^ CFU) in combination with *Str thermophilus* TH-4 is recommended for the prevention of NEC (Szajewska et al., 2023). However, formal quality control reports must be provided to prove viability (van den Akker et al., 2020).

## 5 Conclusion

As bifidobacteria play a pivotal role in infant health and development, leading to long-term reduction of diseases associated with metabolic, immune and neurodevelopment, BB-12 has been extensively investigated for the prevention and treatment of gut dysbiosis in early life. This clinically oriented summary provides a synthesis of the current evidence and highlights key gaps that limit the routine pediatric application of BB-12. The strength of evidence for BB-12 varies substantially across indications, with benefits in infantile colic well established; whereas data for other gut-brain axis disorders and the immune system outcomes remain limited or inconclusive. The effectiveness and safety of BB-12 for the prevention and treatment of preterm infant complications also requires further clarification.

Notably, the current data only support a correlative link between BB-12-induced gut microbiome changes and long-term outcomes. Additional human studies are required to demonstrate a cause-effect correlation between specific changes in gut microbiota and clinical benefits.

Given the absence of specific pediatric guidelines on indications, dosing, and duration, together with the need for rigorous quality control and viability confirmation, further studies are needed.

In addition to the valuable probiotic characteristics displayed by BB-12 (e.g., acid and bile tolerance, adherence properties, pathogen inhibition, enhancement of gut barrier integrity and immunomodulation), it also has beneficial effects on gut microbiome composition, digestive health and gut transit time and regularity (Figure 1). Therefore, it is evident that the use of BB-12 in baby formulas, dietary supplements, and fermented milk products plays a significant role in boosting and maintaining healthy microbiota, potentially leading to long-term health benefits. By distinguishing established benefits from areas of uncertainty, this summary aims to support informed decision-making and define priorities for future research, positioning BB-12 as a promising option for pediatric and neonatal care.
